# Drug-Resistant TB, HIV and COVID-19 Co-Infection: Case Reviews from Kwa-Zulu Natal, South Africa

**DOI:** 10.2147/IDR.S433695

**Published:** 2023-12-15

**Authors:** Razia Hassan-Moosa, Kegaugetswe Wilhemina Motsomi, Radhamoney Narasimmulu, Aida Sivro, Kevindra K Naidu, Ayesha B M Kharsany, Natasha Samsunder, Rubeshan Perumal, Kogieleum Naidoo

**Affiliations:** 1 Centre for the AIDS Programme of Research in South Africa (CAPRISA), Durban, South Africa; 2 MRC-CAPRISA HIV-TB Pathogenesis and Treatment Research Unit, Doris Duke Medical Research Institute, University of KwaZulu-Natal, Durban, South Africa; 3 Department of Medical Microbiology, University of KwaZulu-Natal, Durban, South Africa; 4 JC Wilt Infectious Disease Research Centre, National Microbiology Laboratory, Public Health Agency of Canada, Winnipeg, MB, Canada; 5 Department of Medical Microbiology and Infectious Diseases, University of Manitoba, Winnipeg, MB, Canada

**Keywords:** HIV, DR-TB, COVID-19, case reviews, screening, transmission

## Abstract

**Background:**

Coronavirus disease (COVID-19) potentially exacerbates drug-resistant tuberculosis (DR-TB). We describe the clinical presentation and outcomes of three patients with human immunodeficiency virus (HIV), DR-TB and COVID-19.

**Case One:**

A virologically suppressed 31-year-old man on antiretroviral therapy (ART) and multidrug-resistant (MDR)-TB treatment presented with mild COVID-19 and was hospitalised for 10 days of clinical monitoring, despite being clinically stable with normal baseline inflammatory markers. Severe acute respiratory syndrome coronavirus polymerase chain reaction (SARS-CoV-2 PCR) positivity persisted at Day 28.

**Case Two:**

A virologically suppressed 37-year-old woman on ART and MDR-TB treatment presented with moderate COVID-19. Baseline inflammatory markers were raised, and dexamethasone and azithromycin were initiated with good clinical improvement. SARS-CoV-2 PCR positivity persisted at Day 28.

**Case Three:**

A viraemic 24-year-old woman on second-line ART and MDR-TB treatment, presented with mild COVID-19 disease, normal oxygenation and normal inflammatory markers, and remained clinically stable with negative SARS-CoV-2 PCR at Days 14 and 28.

**Conclusion:**

Screening for SARS-CoV-2 infection is advised for DR-TB patients with new or worsening respiratory symptoms.

## Background

The twin epidemics of HIV and TB continue to challenge South Africa’s public healthcare system, with additional complexity and burden posed by the recent COVID 19 pandemic. Furthermore, South Africa remains a significant contributor to the global incidence of multi-drug resistant TB.^1^ TB co-infection in COVID-19 has been shown to increase the risk of severe COVID-19 disease and poor patient outcomes.^2–4^ A large international multi-centre study found a 15% increased risk of severe COVID-19 disease and 38% risk of mortality from COVID-19 in HIV co-infected patients.^5^ Data on the impact of HIV, DR-TB, and COVID-19 co-infection outcomes in high TB/HIV endemic settings are limited. Local data from the Western Cape has shown a 2-fold increased risk of death in patients with active TB who develop COVID-19 disease [adjusted Hazard Ratio (aHR) 2.70; 95% confidence interval (CI) 1.81–4.04)].^6^ Furthermore, patients with a previous TB history were shown to have an increased risk of death from COVID-19 disease (aHR 1.51; 95% CI, 1.18–1.93).^6^

In addition, overlap of symptoms and clinical presentation of both diseases complicates screening and testing for SARS-CoV-2, especially in patients presenting with acute exacerbations of an existing respiratory condition. Chronic respiratory symptoms could mask new onset symptoms, leading to DR-TB patients not seeking timeous clinical assessment, thereby fuelling SARS-CoV-2 transmission and impeding COVID-19 infection control practices.

A total of 113 participants, 30 HIV-COVID-19 co-infected and 83 HIV-uninfected individuals with COVID-19 were enrolled into the Centre for the AIDS Programme of Research in South Africa (CAPRISA) COVID-19 surveillance study undertaken between July 2020 and March 2021 (BREC/00001195/2020). SARS-CoV-2 PCR testing was conducted on nasopharyngeal swabs collected from patients attending routine health-care services, including ART and TB services. Confirmed COVID-19 cases, who provided informed consent for study participation, were enrolled, and followed up at day 7, 14 and 28 post-screening. All participants were managed by their primary care providers. Radiographic data was accessed from the patients’ medical records. Here, we summarise the clinical disease presentation, clinical management, and outcomes of three HIV, DR-TB, and COVID-19 co-infected patients identified in our cohort.

## Case Reviews

### Case One

**Figure 1:**
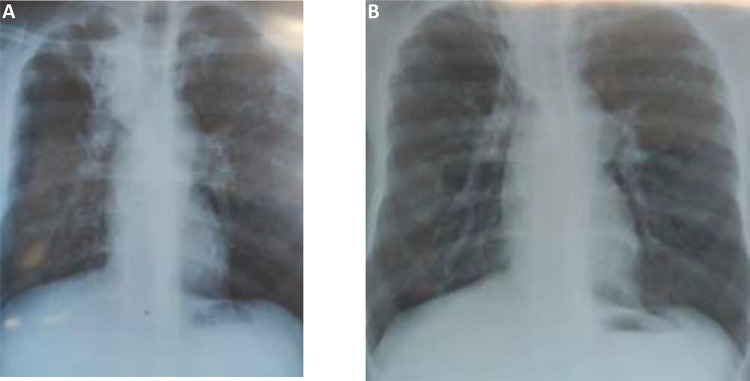
Plain film chest radiographs for patient one - Postero-Anterior view: (**A**) June 2020, showing bilateral upper lobe infiltration and right upper lobe cavity and right paratracheal hilar lymphadenopathy, (**B**) October 2020, showing bilateral upper lobe fibrosis and a residual cavity in left upper lobe.

A 31-year-old unemployed man, receiving ART for HIV, on 5 months of MDR-TB therapy presented for his outpatient clinic visit in August 2020. He reported a one-week history of a new cough, with no known COVID-19 contact. A SARS-CoV-2 PCR was positive, and he was subsequently admitted to the DR-TB/COVID ward for clinical monitoring. He was diagnosed with HIV and a previous episode of DR-TB in 2015. The patient was a known substance abuser who was lost to follow-up from both HIV and TB care on numerous occasions. In March 2020, on re-initiation of MDR-TB therapy, he presented with a CD4+ cell count of 66 cells/microliter (μL) and viral load of 244,004 copies/milliliter (mL). At study enrolment, he reported a three-day history of fever, sore throat, nausea, vomiting and fatigue with partial resolution of cough. He was clinically stable with an oxygen saturation of 100% in room air. His baseline CD4+ cell count was 125 cells/μL, with an undetectable viral load and unremarkable laboratory parameters (Table 1). He had been initiated on a 5-day course of amoxicillin, with resolution of all symptoms on Day 7. He remained asymptomatic and clinically well at subsequent follow-up visits. A chest radiograph taken pre-COVID-19 in June 2020 illustrated infiltrates and cavities in the right and left upper lobes and left lower lobe with associated hilar lymphadenopathy (Figure 1A). Radiological findings two months post COVID-19 depicted bilateral upper lobe fibrosis with a persistent cavity in the left upper lobe (Figure 1B).

**Table 1:** Baseline and Follow-Up Characteristics

Sociodemographic and Clinical Markers	Case 1	Case 2	Case3
Age	31	37	24
Sex (M/F)	M	F	F
Employed/Unemployed	Unemployed	Unemployed	Unemployed
Previous Smoker	Yes	No	No
BMI (kg/m2)	22.8	15	20
ART regimen	TLD*	TLD*	LPV/r/3TC/TDF**
Viral Load (copies/mL)	Not detected	100	577
CD4+ count (cells/µL)	125	243	928
DR-TB resistance profile	Rifampicin Resistant	Rifampicin Resistant	Rifampicin Resistant
DR-TB therapy at the time of COVID-19 diagnosis***	BDQ/CFZ/LVF/ETH/ PAS/DLM	BDQ/PZA/ETH/INH/LVF/CFZ/LZD	CFZ/PZA/ETH/INH/LVF/DLM/PAS/IMP
Sputum AFB smear at time of COVID-19 diagnosis	Negative	Positive	Negative
Previous TB history	Yes	Yes	Yes
Co-morbidities^#^	–	–	–
Current Smoker	Yes	No	No
Known COVID-19 positive contact	No	No	Unsure
**Symptoms at baseline:**			
Fever	No	Yes	No
Cough	Yes	Yes	No
Sore throat	–	–	–
Shortness of Breath	Yes	Yes	Yes
Myalgia	No	No	Yes
Fatigue	No	Yes	No
Loss of Taste	–	–	–
Loss of smell	Yes	No	No
Headache	Yes	Yes	No
Nausea/vomiting	–	–	–
Duration of symptom onset at screening (days)	7	79	2
**Clinical Features at baseline**			
Temperature (°C)	35.9	36.9	36.4
Respiratory Rate (breaths/min)	16	22	19
Oxygen Saturation in room air (%)	100	95	97
**Laboratory Results (normal range):**			
Red Cell count (4.0–5.2x10^12/L)	3.85	2.02	4.66
Haemoglobin (11.1–13.3g/dL)	12.4	**5.5**	12.7
Platelets (180–370 x10˄9)	172	**46**	403
White cell count (4.0–13.0x10˄9)	2.97	4.03	8.82
Neutrophil % (45.5–57.2%)	20.8	**80.2**	49.9
Neutrophil(N) count (2.6–7.3x10˄9)	0.62	3.23	4.40
Lymphocytes% (27.7–45.8%)	52.2	17.1	41.8
Leucocyte(L) count (1.78–3.95x10^9)	1.55	0.69	3.69
N: L ratio	0.4	**4.7**	1.2
d-Dimer (0.0–0.5 µg/mL)	0.21	**3.32**	0.66
CRP (0–5mg/L)	5.0	**54.8**	6.4
Serum creatinine (54.0–110.0µmoL/L)	79.0	39.0	57.0
Bilirubin (0–34 µmol/L)	8	6	6
ALT (10–40 IU/L)	34	23	14
AST (5–40 IU/L)	70	61	26
eGFR (>60 mls/min)	105	170	121
HbA1c (4.0–6.0%)	4.7	5.5	5.3
Hospitalised	Yes	Yes	Yes
**COVID-19 concomitant therapy**			
Supplementary oxygen	–	–	–
Steroids	No	Yes	No
Thrombolytic therapy	–	–	–
Antibiotics	Yes	Yes	No
**COVID-19 Disease severity**			
Baseline	Mild	Moderate	Mild
Day 7	Asymptomatic	Mild	Asymptomatic
Day 14	Mild	Mild	Asymptomatic
Day 28	Asymptomatic	Asymptomatic	Asymptomatic
**SARS-CoV-2 PCR/ Ct**			
Screening (date of screening)	+/ 10.96	+ / 12.41	+/12.32
Baseline	+ / 10.96	+/ 16.80	+/16.85
Day 7	+ / 10.56	+ / 10.48	+/22.23
Day 14	+ / 12.22	+ / 20.79	–
Day 28	+ / 15.20	+ / 30.22	–

**Notes**: *TLD-Tenofovir/Lamivudine/Dolutegravir, **LPV/r-Lopinavir/Ritonavir, 3TC-Lamivudine/TDF-Tenofovir Disoproxil Fumarate, ***BDQ-Bedaquiline, CFZ-Clofazimine, PZA-Pyrazinamide, PAS-Para-Amino Salicylic Acid, DLM-Delamanid, INH-Isoniazid, IMP-Imipenem, ETH-Ethionamide, LVF-Levofloxacin, LZD-Linezolid. ^#^Co-morbidities include Diabetes, Hypertension, COPD, Cardiac Disease, Asthma. Values in bold are out of range.

Plain film chest radiographs for patient one - Postero-Anterior view: (A) June 2020, showing bilateral upper lobe infiltration and right upper lobe cavity and right paratracheal hilar lymphadenopathy, (B) October 2020, showing bilateral upper lobe fibrosis and a residual cavity in left upper lobe.

### Case Two

**Figure 2:**
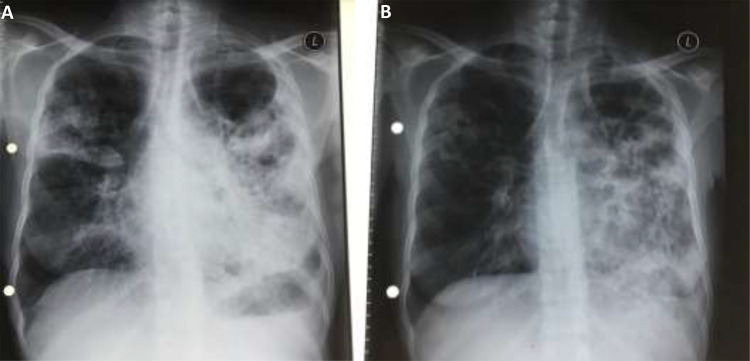
Plain film chest radiographs for patient two - Postero-Anterior view: (**A**) September 2020, showing extensive bilateral infiltration and cavitation, involving the left lung more extensively than the right, with left upper lobe bullous lesion (**B**) March 2021, showing fibrosis of the right upper lobe with residual cavities, and fibro-cavitary changes involving of the left lung.

A 37-year-old woman with known HIV/MDR-TB co-infection, who was lost to follow-up from both HIV and TB care, was admitted in September 2020 for re-initiation of ART and MDR-TB therapy. At the time of admission, she was noted to be emaciated, with a CD4+ cell count of 222 cells/μL and viral load of 1796 copies/mL. Two months later, while still admitted, she was observed to be acutely ill, with new onset dyspnoea, tachypnoea, and fatigue, with an oxygen saturation of 95% in room air. She subsequently tested positive for COVID-19. On examination, she had reduced air entry in the left chest and was assessed as having moderate COVID-19 disease. Baseline laboratory markers indicated an elevated C-reactive protein (CRP) of 54.8 mg/L, d-dimer of 3.32 ug/mL, a neutrophil-to-lymphocyte ratio of 4.7, severe anaemia and thrombocytopenia, a CD4+ cell count of 243 cells/μL and viral load of 100 copies/mL (Table 1). Linezolid was stopped due to anaemia and she received three units of blood. She was initiated on dexamethasone (6mg daily) and azithromycin (500mg daily) for moderate-severe COVID-19. On Day 7 of follow-up, the patient reported new onset myalgia, loss of taste, nausea, and vomiting. Her dyspnoea had resolved and her oxygen saturation improved to 98% in room air; laboratory markers had improved with haemoglobin of 7.6 g/dL, platelets of 329 x10^9^/L and d-dimer of 1.25mg/L with the exception of an elevated CRP of 114 mg/L. At subsequent follow-up visits, clinical improvement with resolution of all symptoms was noted, however no confirmation of improvement in inflammatory markers was available. She continued to receive in-patient MDR-TB therapy. A chest radiograph taken in September 2020 illustrated bilateral consolidation and cavitation, involving the left lung more extensively than the right, with a large bullous lesion in the left lung (Figure 2A). Two months post-COVID-19, chest radiography demonstrated right upper lobe fibrosis with extensive left lung fibro-cavitary changes (Figure 2B).

### Case Three

**Figure 3:**
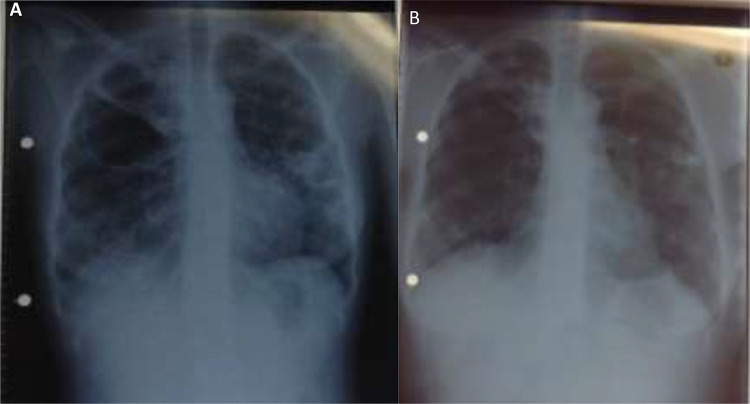
Plain film chest radiographs for patient three - Postero-Anterior view: (**A**) July 2020, showing right upper, mid-, and lower zone and left lower lobe consolidation and cavities in right upper and left lower lobes and right hilar lymphadenopathy. (**B**) January 2021, showing bilateral lower lobe fibrosis with fibro-cavitary changes of the right upper lobe.

A 24-year-old woman receiving ART for HIV, who was lost to follow-up from MDR-TB care, was admitted in July 2020 for re-initiation of MDR-TB therapy. At the time of admission, she was on a second-line ART regimen comprising of lopinavir/ritonavir, tenofovir, and lamivudine with a CD4+ cell count of 1224 cells/μL and viral load of 18,418 copies/mL. She had been clinically stable throughout the period of admission and was given a temporary discharge in December 2020. She returned for re-admission 2 weeks later and complained of a two-day history of new-onset shortness of breath and fatigue. SARS-CoV-2 was detected by PCR on a nasopharyngeal swab. She was clinically stable at both screening and enrolment visits, with room air oxygen saturations of 96% and 97%, respectively. Her baseline laboratory findings were unremarkable, with a CD4+ cell count of 928 cells/μL and viral load of 577 copies/mL. She remained asymptomatic and clinically well at all follow-up visits. She did not receive any COVID-19 specific therapy. Chest radiological findings noted prior to initiation of MDR-TB therapy included bilateral consolidation and cavitation (Figure 3A). Improved radiological findings were noted approximately two-week post COVID-19 with bilateral lower lobe fibrosis and fibro-cavitary changes in the right upper lobe (Figure 3B).

## Discussion

We describe two cases of mild and one case of moderate-severe COVID-19 in patients with DR-TB/HIV co-infection. Despite pre-existing DR-TB lung pathology, all three patients had an unremarkable clinical course of COVID-19 with favourable outcomes, showing that in otherwise healthy individuals, below the age of 60, appropriately managed DR-TB co-infection does not seem to worsen COVID-19 outcomes. Although our numbers are small, these observations are consistent with data from other cohorts looking at younger people with few or no co-existing comorbidities.^7^,^8^ While several other studies have shown that patients with active TB infection are at an increased risk of mortality and morbidity, the majority of the patients in these studies had several other risk factors associated with poor COVID-19 outcomes including older age and the presence of other comorbidities.^7^,^8^ Data describing severe COVID-19 disease in patients with drug susceptible TB (DS-TB) emanating from China,^9^ Singapore^10^ and Saudi Arabia^11^ identified hypertension, diabetes, and older age as significant risk factors for severe COVID-19 disease, whereas reports from HIV endemic settings report HIV co-infection as a risk factor for in-hospital COVID-19 related mortality.^12^ Despite HIV viraemia being identified as an additional risk factor for severe COVID-19 in TB/HIV co-infected patients in a case report from Panama,^13^ individuals with controlled HIV infection on ART are noted to have no greater risk of severe COVID-19 disease compared to HIV-negative patients.^14^ Immune suppression has been cited as a risk factor for severe COVID-19 disease,^15^ however this was not observed in the two patients in our study with low CD4+ cell counts, both of whom presented with mild disease.

Interestingly, the two patients with significantly compromised immunity remained SARS-CoV-2 positive at day 28 of follow-up. These data are consistent with recent reports of prolonged SARS-CoV-2 viral shedding in immunocompromised patients.^16^ Prolonged viral shedding may fuel SARS-CoV-2 transmission and has been associated with the accumulation of intra-host SARS-CoV-2 mutations in patients with advanced HIV^17^ and reduced SARS-CoV-2 specific IgG and IgM antibody responses.^18^ Incidentally, patient two who reported a prolonged history of respiratory symptoms remained persistently sputum acid fast bacillus (AFB) positive, with significant radiological findings noted four months post-COVID-19 disease. Scientific literature remains scarce on SARS-CoV-2 viral kinetics and disease evolution in patients co-infected with DR-TB. A retrospective analysis of six newly diagnosed DS-TB/COVID-19 co-infected patients described prolonged viral shedding beyond 4 weeks, in 50% of patients.^17^ Patients were also noted to have delayed sputum conversion, however none were HIV co-infected.^19^ The impact of active TB replication and TB disease on SARS-CoV-2 viral kinetics needs further exploration and understanding.

Finally, our case series highlights the need to integrate routine screening and testing for SARS-CoV-2 within in-patient and out-patient DR-TB services. COVID-19 symptom screening was offered to all in-patients and out-patients using an opt-out approach, while testing was offered to those with symptoms or a known high-risk exposure with a COVID-19 infected person. Patient one accessed testing on his own volition. Parallel testing for both TB and SARS-CoV-2 infection should be standard of care in TB endemic settings. Maintaining a high index of suspicion for SARS-CoV-2 infection is essential for all patients irrespective of the pre-existing diagnosis, especially in those presenting with new or exacerbated respiratory symptoms.

## Limitations

Our findings are restricted to HIV-MDR-TB coinfected patients presenting with mild-to-moderate COVID-19 disease and do not describe clinical manifestations of severe COVID-19 disease in this population. Access to radiological services for infectious COVID-19 patients was limited due to strict COVID-19 infection control practices. No radiological investigations were undertaken to guide COVID-19 disease management as all three patients did not present with or progress to severe disease. Repeat testing for inflammatory markers, HIV viral load and CD4+ cell count measurements were not undertaken at follow-up visits.

## Conclusion

Our report highlights the importance of parallel screening and testing for SARS-CoV-2 in people with TB, given the overlap of symptoms, and especially given the risk of COVID-19 being overlooked in patients with chronic respiratory symptoms. We therefore recommend routine and regular screenings for SARS-CoV-2 at all in-patient and out-patient TB service points in TB endemic settings, irrespective of the duration of TB therapy. Subtle changes in clinical presentation or engagement or re-engagement of patients into care should be triggers for SARS-CoV-2 testing. This will enable rapid diagnosis, isolation, and management of COVID-19. Further research into SARS-CoV-2 viral kinetics as well as antibody responses in individuals co-infected with TB, including DR-TB, is warranted to guide COVID-19 disease management, interventions to mitigate transmission, and COVID-19 vaccination programmes for patients with active TB disease, especially in high TB/HIV burden settings.

## Data Sharing Statement

All data generated or analysed during this study are included in this published article.
